# Topological Data Analysis for Eye Fundus Image Quality Assessment

**DOI:** 10.3390/diagnostics11081322

**Published:** 2021-07-23

**Authors:** Gener José Avilés-Rodríguez, Juan Iván Nieto-Hipólito, María de los Ángeles Cosío-León, Gerardo Salvador Romo-Cárdenas, Juan de Dios Sánchez-López, Patricia Radilla-Chávez, Mabel Vázquez-Briseño

**Affiliations:** 1 Facultad de Ingeniería Arquitectura y Diseño, Universidad Autónoma de Baja California, Carretera Transpeninsular Ensenada-Tijuana #3917, Playitas, Ensenada 22860, Mexico; romo.gerardo@uabc.edu.mx (G.S.R.-C.); jddios@uabc.edu.mx (J.d.D.S.-L.); mabel.vazquez@uabc.edu.mx (M.V.-B.); 2 Dirección de Investigación, Innovación y Posgrado, Universidad Politécnica de Pachuca, Carretera Ciudad Sahagún-Pachuca Km. 20, Ex-Hacienda de Santa Bárbara, Hidalgo 43830, Mexico; ma.cosio.leon@upp.edu.mx; 3 Escuela de Ciencias de la Salud, Universidad Autónoma de Baja California, Carretera Transpeninsular S/N, Valle Dorado, Ensenada 22890, Mexico; patyradilla@uabc.edu.mx

**Keywords:** persistent homology, eye fundus images, topological data analysis, image quality assessment, computational ophthalmology

## Abstract

The objective of this work is to perform image quality assessment (IQA) of eye fundus images in the context of digital fundoscopy with topological data analysis (TDA) and machine learning methods. Eye health remains inaccessible for a large amount of the global population. Digital tools that automize the eye exam could be used to address this issue. IQA is a fundamental step in digital fundoscopy for clinical applications; it is one of the first steps in the preprocessing stages of computer-aided diagnosis (CAD) systems using eye fundus images. Images from the EyePACS dataset were used, and quality labels from previous works in the literature were selected. Cubical complexes were used to represent the images; the grayscale version was, then, used to calculate a persistent homology on the simplex and represented with persistence diagrams. Then, 30 vectorized topological descriptors were calculated from each image and used as input to a classification algorithm. Six different algorithms were tested for this study (SVM, decision tree, k-NN, random forest, logistic regression (LoGit), MLP). LoGit was selected and used for the classification of all images, given the low computational cost it carries. Performance results on the validation subset showed a global accuracy of 0.932, precision of 0.912 for label “quality” and 0.952 for label “no quality”, recall of 0.932 for label “quality” and 0.912 for label “no quality”, AUC of 0.980, F1 score of 0.932, and a Matthews correlation coefficient of 0.864. This work offers evidence for the use of topological methods for the process of quality assessment of eye fundus images, where a relatively small vector of characteristics (30 in this case) can enclose enough information for an algorithm to yield classification results useful in the clinical settings of a digital fundoscopy pipeline for CAD.

## 1. Introduction

### 1.1. Public Health Dimension

Eye health has a profoundly multidimensional effect in overall health, economics, and social development for populations around the world [1]. Globally, there are more than 250 million people with vision impairment and over a billion with near-vision impairment [2]. It is projected that over the next 30 years, the amount of people affected by these issues will triple, reaching around 700 million, mostly due to the aging and growth of the population [2]. Even more, 90% of this loss occurs in low-income and middle-income countries leading to a substantial economic burden with a global annual estimate of over USD 3 trillion [3].

Even though diagnostic and therapeutic strategies are available for the many causes of sight loss, for a significant amount of the global population at risk, they remain inaccessible mainly due to the lack of local eye care services and a considerable shortage and inefficient distribution of appropriately trained personnel [4].

To address these challenges, with enough evidence and with potential to scale, these strategies can focus on capacity building of clinical personnel and the use of technology to empower human resources [5]. Computational approaches in digital image analysis have been proposed as a strategy to strengthen and complement eye health teams, facilitating accessibility to health services for medically underserved populations, one of the areas in which computational tools have proved to be useful is that of digital image processing as computer-aided diagnosis (CAD) systems [6].

A fundamental aspect in the evaluation of eye health is the assessment of eye fundus through ocular fundoscopy [7].

This can be achieved by direct observation of the eye fundus through an ophthalmoscope or through eye fundus images. As the methodology to evaluate a fundoscopy is not universal, it is strongly recommended that a systematic and organized approach be taken to this approach in the clinical practice [8]. This will allow this approach to be adapted and reproduced with a computational system that mimics the evaluations a clinician olud perform during a regular consultation.

Nonetheless, state-of-the-art algorithms used for CAD systems in eye fundus images [9,10,11] tend to need large volumes of images in their training stages in order for them to achieve acceptable performance indicators, as well as accurately labeled images [12]. Therefore, the exploration of approaches to produce robust results with a relatively smaller volume of training images is important, given the current tendency in the field towards the use of deep learning approaches that require significantly large annotated datasets, which is a current challenge in medicine [13]. This will allow CAD tools to adjust to a clinical environment more rapidly, thus, facilitating expected outcomes.

### 1.2. Fundus Image Analysis

**Figure 1:**
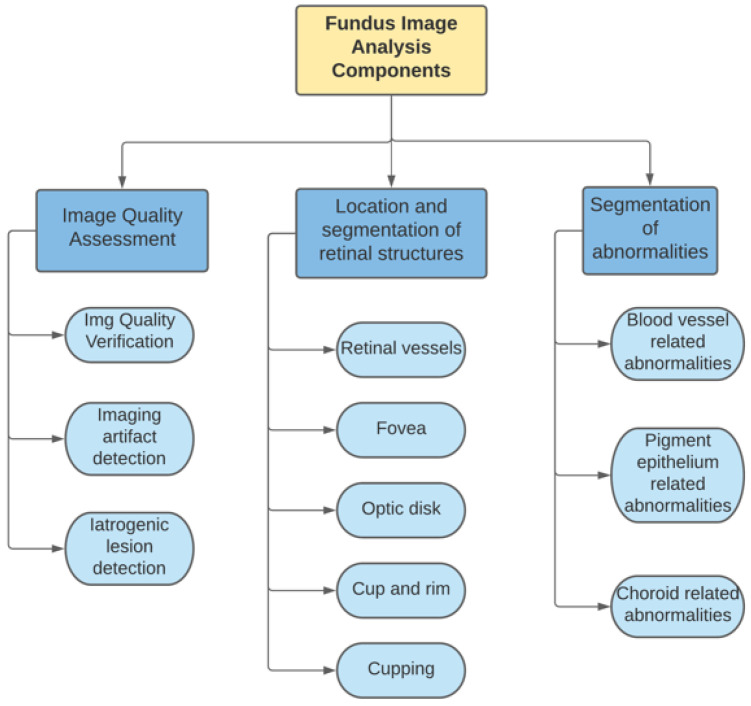
Fundus image analysis components as proposed by [14].

Fundus image analysis can be understood as the process of obtaining a digital image of the eye fundus and the analytical pipeline required to generate a CAD tool to support physicians in their clinical practice [14,15]. Eye fundus imaging is the most established technique of retinal imaging; Figure 1 shows a summary of the components considered for this process.

As proposed by Abràmoff [14], image quality assessment (IQA) is considered the first step in automated analysis techniques of eye fundus images. Development of IQA algorithms depends on the clinical application of the overall analysis [16,17,18] and can be classified in three general groups according to the techniques in which they achieve their objective:

1. Image quality parameters.

These algorithms are based on parameters like clarity, focus, contrast, and illumination. They generally have low computational complexity and are preferred when using mobile or low powered devices, some examples can be seen in [19,20] and [21].

2. Based on segmentation.

Techniques such as image structure clustering [22] or segmentation maps and feature analysis fall on this category [23].

3. Deep learning.

The most recent approach in IQA, done with convolutional neural networks (CNNs) [9] and human-in-the-loop approaches combined with CNNs [24].

### 1.3. Topological Data Analysis

Topology mainly focuses on the analysis of the characteristics preserved under continuous deformation in geometric objects [25] (for example, noise or missing data represented in point clouds). In recent years, there has been a significant rise in computational approaches to topological concepts for the analysis of data [26,27,28]; this area is known as topological data analysis (TDA).

For the analysis of medical data, TDA becomes an attractive tool given the robustness of the methods to missing and noisy data, which are common in this domain.

In particular, for the case of images, they can be interpreted into mathematical objects called cubical simplicial complexes and then analyzed with topological methods to obtain characteristics to be used later in the analytical pipeline (Section 3.2). In this work, we focus on the interpretation of eye fundus images as cubical simplicial complexes and the obtention of homological groups (Betti numbers, $\beta_{\left\{1,2\right\}}$) to be used in the process of selecting optimal threshold values for binarizing a grayscale version of the image to obtain masks for background segmentation.

## 2. Materials and Methods

**Figure 2:**
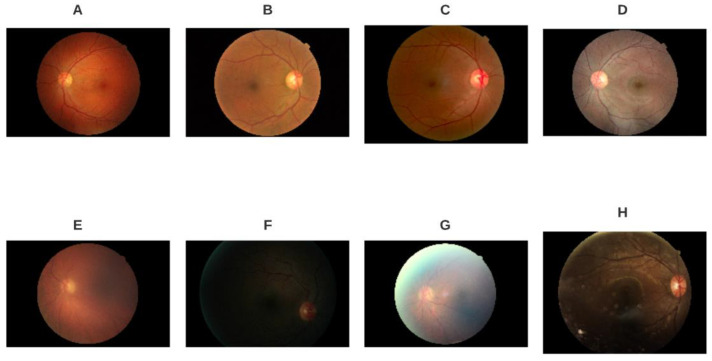
Examples of EyePACS images selected for the study with label good quality (**A**–**D**) and bad quality (**E**–**H**). Notice the variability within labels given by color, illumination, blurring, and anatomical landmarks.

Eye fundus images used for this project were taken from the EyePACS project [29]. For the particular task of image quality assessment, we used labels proposed in [30], as presented by [31]. We modeled the phenomenon as a binary classification between images with quality (good) and those without quality (bad) and also adjusted for class imbalance by randomly selecting subsets by label of 2000 images each. Figure 2 shows a sample of images by label.

## 3. Methods

### 3.1. Topological Interpretation of Digital Images

#### 3.1.1. Cubical Complexes for the Representation of Digital Medical Images

**Figure 3:**
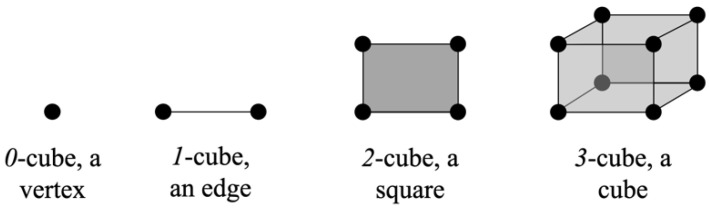
Visual summary of the general process for topology-regulated background extraction of eye fundus digital images.

Considering the need to use mathematical objects to represent 2D digital images, cubical complexes arise as a natural representation for this domain [32] given that pixels can be represented by *2-*cubes and voxels by *3-*cubes, as shown in Figure 3. This approach has already been used by [33] to analyze images of blood vessels by interpreting segmented images from magnetic resonance imaging as cubical simplexes and calculating their homological groups.

A cubical complex is a combinatorial structure used in topological analysis where a *0-*cube is called a vertex, a *1-*cube, an edge, a *2-*cube a square, and a *3-*cube, a cube. This concept is built on the theory of elementary intervals and cubes; an in-depth approach to the theory of cubical complexes is presented by [34].

In a cubical complex (*K*), multidimensional cubes ($\sigma^{d}$) play the role of simplices, where a finite cubical complex in ${\mathbb{R}}^{d}$ is a union of cubes aligned on the grid ${\mathbb{Z}}^{d}$ satisfying specific conditions as with simplicial complexes.

A *d-*dimensional digital image can be considered as a discrete map $ℐ:I\subseteq {\mathbb{Z}}^{d}\to \mathbb{R}$. In this context, an element v∈I is called a pixel when *d = 2* and a voxel when *d = 3*. ℐ(𝓋) is the correspondent intensity or grayscale value. For the case of a binary image, the discrete map is $ℬ:I\subseteq {\mathbb{Z}}^{d}\to \left\{0,1\right\}$.

For the purpose of this work, we choose to represent images as cubical complexes where a pixel is represented by a *d-*cube, and all its faces (adjacent lower-dimensional cubes) are added. A function on the resulting cubical complex *K* by extending the values of the pixels to all the cubes σ in *K* as the next formula shows:

(1) $$ℐ\prime \left(\sigma \right):=\underset{\sigma \;\mathrm{face}\;\mathrm{of}\;\tau }{\min}ℐ\left(\tau \right)$$

Therefore, a grayscale image comes with a natural filtration embedded in the grayscale values of its pixels. Let *K* be the cubical complex built from the image *I*. Then, let:

(2) $$K_{i}:=\{\sigma \in K|ℐ\prime \left(\sigma \right)\leq i\}$$

for the *i*-th sublevel set of *K*. The set ${\left\{K_{i}\right\}}_{i\in ℐ}$ defines a filtration of cubical complexes, indexed by the value of the grayscale function ℐ.

All images in the EyePACS dataset are in the 8-bit format; therefore, the grayscale versions have a depth of luminescence of 256 values (0 for absolute black, 255 for absolute white, and shades of gray for the corresponding values in between them). According to the previous information and following Formula (2), a natural filtration with 256 sublevels can be extracted from these images once they are interpreted as *2-*cubical simplices given that i={0,1,2,…,256}.

**Figure 4:**
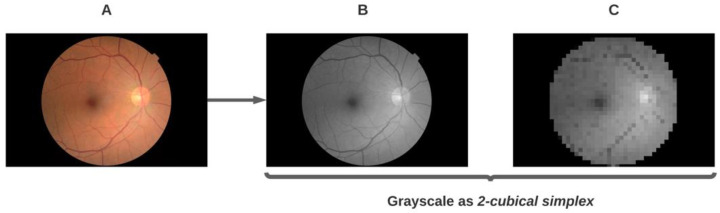
Example of the process of transforming a color eye fundus image from the EyePACS dataset (**A**) to the grayscale version (**B**) and a simplification to show how pixels in a grayscale image fulfill the definition of a cubical complex in 2 dimensions (**C**).

Figure 4C exemplifies the issue of a pixelated version of Figure 4B where pixels are visible as cells in a 2D grid (the image matrix) and naturally interpretable as *2-*cubes (see Figure 2), each one of the four sides of a pixel (lines delimiting each cell in the image matrix) can be interpreted as a *1-*cube (see Figure 2). Following a combinatorial approach, we obtain a mathematical object representing the digital grayscale image on which topological data analysis can be performed, namely the 2D cubical complex.

#### 3.1.2. Cubical Filtrations

The steps to obtain a filtration from a grayscale image are then:

Image→Cubical complex→Sublevel sets→Filtration.

**Figure 5:**
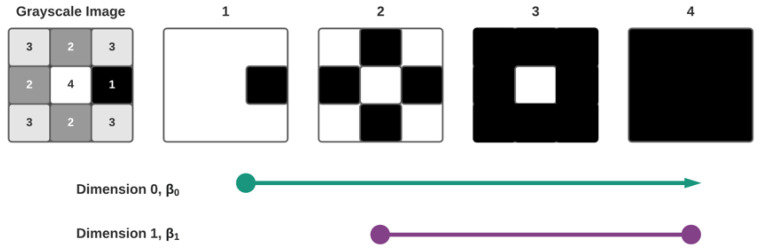
Induced filtration of a 3×3 grayscale image matrix and the correspondent barcode for $\beta_{0}$ and $\beta_{1}$. Using a luminescence depth of 4 elements, L={1,2,3,4}, to simplify the example.

**Figure 6:**
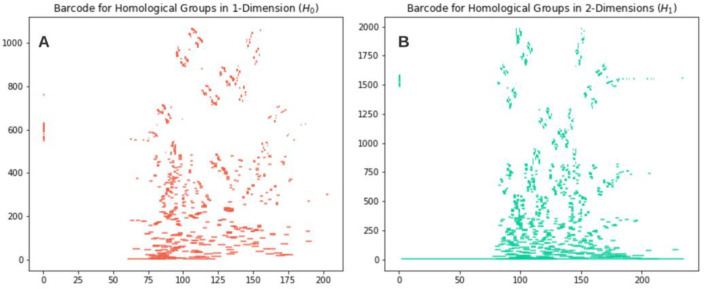
Corresponding bar codes for *0* and *1-*dimensional homological groups (**A**,**B**) of Figure 4B. Displayed on the *x* axis are the filtrations sublevels (since it is an 8-bit image there are 256 sublevels available). The *y* axis shows a count of structures per sublevel in the form of bar codes. Each barcode starts and finishes at the birth and dead sublevel value for the structure it represents.

Figure 5 shows a filtration of a cubical complex induced by the grayscale values contained in the image matrix and the corresponding barcode; notice that the barcode only has one element per homological group (0 and 1 dimensional topological characteristics). This information is reported in the form of Betti numbers ($\beta_{i}$); where $\beta_{0}=$ the number of *0-*dimensional topological characteristics or connected components (connected complete black pixels for the use case of this work) at a specific sublevel filtration, $\beta_{1}=$ the number of 1-dimensional topological characteristics or holes inside connected components (complete white pixels inside components made of black pixels). This analysis is called persistent homology (PH) [35]. The figure presents an oversimplified example. Real data will typically have multiple bars per homological group in a filtration as seen in Figure 6.

In the example shown in Figure 5, only one connected component appears at filtration time 1 and survives until the end, as all the new pixels are always connected to the previous ones. A *1-*dimensional homological group ($H_{1}$) appears at filtration time 2 and is filled in at filtration time 4. Figure 6 presents barcodes for the grayscale eye fundus image shown in Figure 4B. As it can be appreciated, there is a significant quantity of barcodes with birth and death values close to each other (short bars) in both $H_{0}$ and $H_{1}$. The structures represented by the short bars in the barcode are considered topological noise and are usually discarded. Longer bars represent structures, connected components for $H_{0}$ or holes for $H_{1}$, that persist through a significant amount of filtration sublevels and could hold information relevant for processes in the clinical diagnosis.

**Figure 7:**
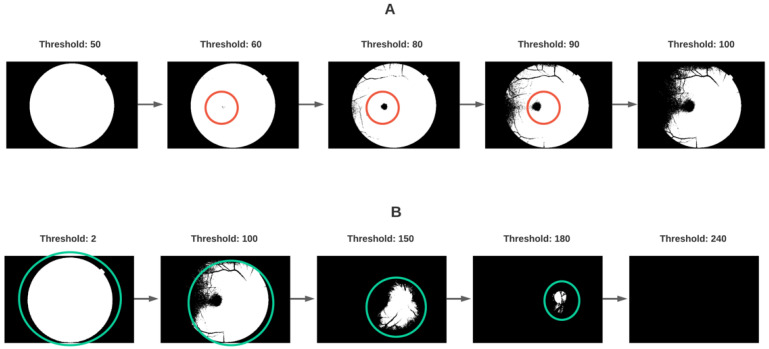
Visualization of binarized images from Figure 4B thresholded at grayscale values contained in the topological elements persisting for the largest amount of sublevel sets in the filtration represented by barcodes in Figure 6. Done for the bar in $H_{0}$ (**A**) and $H_{1}$ (**B**).

Figure 6A,B shows at least one bar that persists longer than the rest for each dimension ($H_{0}$ and $H_{1}$). Figure 7 explores in a visualization some of the sublevel sets (grayscale values) corresponding to each bar per dimension.

By binarizing Figure 4B using threshold values contained in the longest bar of Figure 6A, we can appreciate the resulting series in Figure 7A; a connected component of clinical relevance appears at around a grayscale value of 60 and persists until a value of 90 before merging into a larger unified component with the background at around a grayscale value of 100. Interestingly this spot, connected component, or *0-*dimensional homological group is consistent with the macula and fovea as anatomical landmarks. It is here at this point that the topological results begin to connect with clinical meaning for the domain from which the data come, in this case eye fundus images and the diagnostic process in which they are used.

Figure 6B also shows a *1-*dimensional homological group persisting significantly more than the rest in the filtration (from 2 to over 200). This means that there must be a recognizable hole ($H_{1}$ homological group) when binarizing the image using threshold values contained in the bar for such homological group. Figure 7B shows a series of binarized images using some of the values in the range of 2 to 240 and visually confirms the topological findings with a persistent hole, which first represents all the area of the camera sensor in which the information of the eye fundus image is contained and shrinks until the anatomical region where the optic disk would be expected to be. This is consistent with clinical practice where the area of the optic disk is typically regarded as the brightest in a healthy eye fundus evaluation, again, connecting *1-*dimensional topological structures to clinical meaning.

### 3.2. Topological Indicators Derived from Digital Images

Once an eye fundus image has been interpreted as a 2D cubical complex and a corresponding PH analysis has been performed, as shown in Section 3.1. The results must be vectorized to provide an input that machine learning (ML) algorithms can take for classification purposes. For this work. ML algorithms are used to perform image quality assessment of the eye fundus images. This section presents details dealing with the process of vectorization of topological results represented by persistence diagrams.

**Figure 8:**
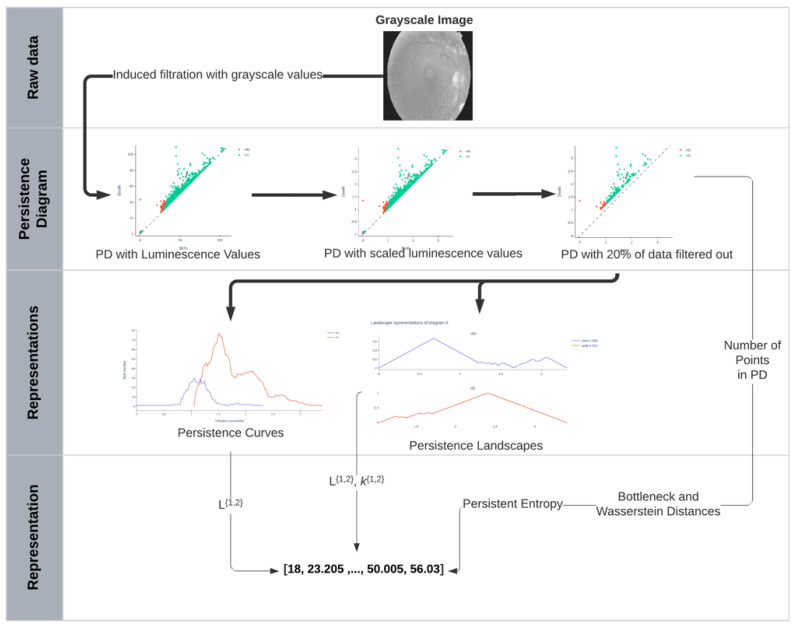
Representation of pipeline for extraction of topological descriptors of eye fundus images.

Figure 8 shows a representation of the pipeline used in this work to perform topological data analysis (TDA) and obtain a vectorized representation to feed a ML classifier (in this case a logistic regression). Each eye fundus image was first converted from color to grayscale (Figure 4) and, then, entered the pipeline described in this section.

#### 3.2.1. Persistence Diagrams

A persistence diagram (PD) is a visual representation of a set of points {(b,d)|b,d∈ℝ²} and d>b, where *d* = death of the topological feature and *b =* birth of the topological feature [36]. In order to be able to feed topological features represented in a persistent diagram to a machine learning algorithm they must be vectorized.

**Figure 9:**
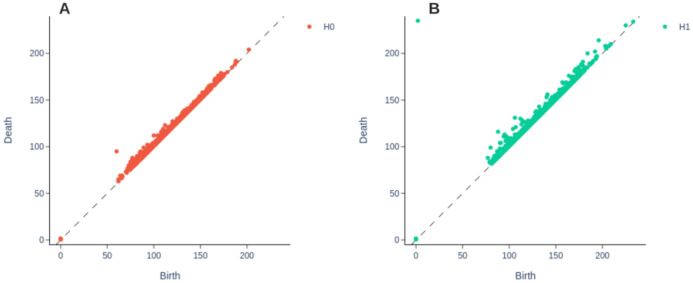
Persistence diagrams (PDs) corresponding to barcodes in Figure 6. Notice that the same information is presented in a more understandable manner, where the diagonal line represents the birth value and the points above the diagonal represent the dead value of a given topological characteristic. PDs facilitate the identification of noise versus relevant topological characteristics.

Figure 9 shows the corresponding PD for *0* and *1*-dimensional topological characteristics obtained from a PH analysis of Figure 4B. Notice that the results are consistent with those observed in the code bars in Figure 6, only that it is easier to identify persistent characteristics.

The following paragraphs explain how results shown in a PD are vectorized for the analysis implemented in this work. From this point on, eye fundus images are represented by their corresponding PDs over which the rest of the analysis is performed.

#### 3.2.2. Persistent Entropy of Persistence Diagrams

It is an intuitive measure of the entropy of the points in a persistence diagram. It results from extracting the Shannon entropy of the persistence (lifetime) of all cycles (topological structures) [37,38].

Let $D={\left\{\left(b_{i},d_{i}\right)\right\}}_{i\in I}$ be a persistence diagram with each $d_{i}<+\infty$, where $b_{i}$ = the *i*-th topological birth point of the structure, and $d_{i}$ = the *i*-th topological dead point of the structure. The persistence entropy of *D* is defined by:

(3) $$PE\left(D\right)=\sum_{i=1}^{n}\frac{l_{i}}{L\left(B\right)}\log\left(\frac{l_{i}}{L\left(B\right)}\right)$$

where $L\left(B\right):=l_{1}+,\ldots +l_{n}$ and $L\left(B\right):=l_{1}+,\ldots +l_{n}$.

#### 3.2.3. Bottleneck Distance

Let *X* and *Y* be two persistence diagrams. To define the distance between them, we consider bijections η:X→Y and record the least upper bound (*sup*) of the distances between corresponding points for each.

Measuring distance between points $x=\left(x_{1},x_{2}\right)\;\mathrm{and}\;y=\left(y_{1},y_{2}\right)$ with $L_{\infty }-\mathrm{norm}:\;\|x-y{\|}_{\infty }=\max\left\{\left|x_{1}-y_{1}\left|,\right|x_{2}-y_{2}\right|\right\}$ and taking the greatest lower bound (*inf*) over all bijections, we get the bottleneck distance between diagrams [39], as shown in Equation (4).

(4) $$W_{\infty }\left(X,Y\right)=\underset{\eta :X\to Y}{inf}\underset{x\in X}{sup}\|x-\eta \left(x\right){\|}_{\infty }$$

A drawback of the bottleneck distance is that it is insensitive to details of the bijection beyond the furthest pair of corresponding points.

#### 3.2.4. *p*-Wasserstein Distance

The *p-*Wasserstein distance between *X* and *Y* for any positive real number *p*, takes the sum of *p*-th powers of the $L_{\infty }$ distances between corresponding points, again minimizing over all bijections, as shown in Equation (5).

(5) $$W_{p}\left(X,y\right)={\left[\underset{\eta :X\to Y}{\inf}\underset{x\in X}{\sum }\;\|x-\eta \left(x\right){\|}_{\infty }^{q}\right]}^{1/p}$$

It is also known as the Earth’s movers distance, because intuitively, it can be interpreted as the minimum energy cost of moving and transforming a pile of dirt in the shape of one probability distribution to the shape of the other distribution. Therefore, the *q-*Wasserstein distance measures the similarity between two persistence diagrams using the sum of all edge lengths [40].

#### 3.2.5. Persistence Landscape

The *k*-th persistence landscape of a barcode ${\left\{\left(b_{i},d_{i}\right)\right\}}_{i=1}^{n}$ in the function $\lambda_{k}:\mathbb{R}\to \left[0,\infty \right)$ is the *k*-th largest value of ${\left\{f_{\left(b_{i},d_{i}\right)}\left(x\right)\right\}}_{i=1}^{n}$, with:

(6) $$f_{\left(b,d\right)}\left(x\right)=\{\begin{array}{c} 0\;if\;x\in \left(b,d\right)\\ x-b\;if\;x\in \left(b,\frac{b+d}{2}\right)\\ -x+d\;if\;x\in \left(\frac{b+d}{2},d\right) \end{array}$$

The parameter *k* is called the layer. In this work, we consider curves obtained when k∈{1,2} [41,42].

#### 3.2.6. Betti Curves

For the purpose of this work, the Betti curve $B_{n}:I\to \mathbb{R}$ of a barcode $D={\left\{\left(b_{i},d_{i}\right)\right\}}_{j\in I}$ is the function that returns for each step i∈I, the number of bars $\left(b_{j},d_{j}\right)$ that contain *i*, as shown in Equation (7).

(7) $$i\mapsto \#\left\{\left(b_{j},d_{j}\right),i\in \left(b_{j},d_{j}\right)\right\}$$

where # stands for cardinality.

#### 3.2.7. Gaussian Kernel

By placing Gaussians of standard deviation σ over every point of the persistence diagram and a negative Gaussian of the same standard deviation in the mirror image of the points across the diagonal, the output of this operation is a real-valued function on ${\mathbb{R}}^{2}$. For this work, we use σ∈{1.6,3.2} [43].

#### 3.2.8. Number of Points in Persistence Diagram

This indicator refers to the number of off-diagonal points in a given persistence diagram, per homology dimension.

Given a persistence diagram consisting of birth–death–dimension triples [b,d,q], subdiagrams corresponding to distinct homology dimensions are considered separately, and the respective numbers of off-diagonal points are counted and reported as the result.

### 3.3. Machine Learning Classifiers

Once the topological indicators are vectorized as mentioned in Section 3.2, the dataset is ready to be processed by standard machine learning classifiers.

For this work, we explored the following algorithms:

- Support vector machine;
- Classification tree;
- k-nearest neighbors;
- Random forest;
- Logistic regression;
- Multilayered perceptron.

For these initial explorations, the authors used Orange3 [44], selecting the default hyperparameters proposed by the software for each of the algorithms. Then, a subset of algorithms was selected based on their performance and evaluated in more depth in order to choose the classifier to use for this work.

### 3.4. Metrics for Evaluation of Performance of Classification Algorithms

**Figure 10:**
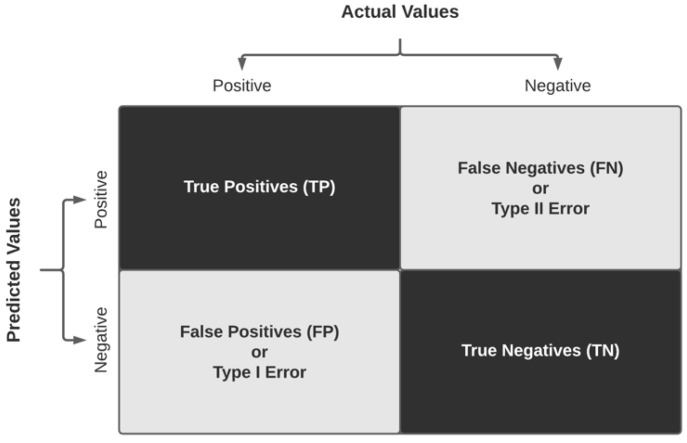
Visualization of a generic confusion matrix.

Given a confusion matrix, as shown in Figure 10, in the context of a binary classification, the following indicators are identified:

- **True positives (TP):** entities classified by the algorithm as true to the label evaluated when the reference is also true.
- **True negatives (TN):** entities classified by the algorithm as true to the label evaluated when the reference is false.
- **False positives (FP):** entities classified by the algorithm as false to the label evaluated when the reference is also false, also known as Type I Error.
- **False negatives (FN):** entities classified by the algorithm as false to the label evaluated when the reference is true, also known as Type II Error.

The following metrics are used to report on algorithm performance on this work [45]:

- Accuracy:

This metric answers the following question: overall, how often is our model correct? It is calculated using Equation (8):

(8) $$Accuracy=\frac{TP+TN}{TP+TN+FP+FN}$$

It does not work well with class imbalance, nor does it give detailed information about the application of the problem.

- Precision:

This metric answers the following question: what is the ratio between the true positives and all the positives? It is calculated using Equation (9):

(9) $$Precision=\frac{TP}{TP+FP}$$

This metric helps when the impact (defined by the domain of the data) of false positives is significantly high.

- Recall:

This metric answers the following question: what is the measure of our model correctly identifying true positives? It is calculated using Equation (10):

(10) $$Recall=\frac{TP}{TP+FN}$$

This metric helps when the impact (defined by the domain of the data) of false negatives is significantly high. It is also known as sensitivity or true positive rate (TPR).

- F1-score:

This metric is a combined measure of precision and recall. Therefore, a relatively high F1-score will mean that there are low false positives and low false negatives in the results generated by the classifier. It is calculated using Equation (11):

(11) $$F1=2\times \frac{\frac{TP}{TP+FP}\times \frac{TP}{TP+FN}}{\frac{TP}{TP+FP}+\frac{TP}{TP+FN}}=2\times \frac{precision\times recall}{precision+recall}$$

- Receiver-operating characteristic (ROC) curve:

This curve is considered a comprehensive performance measure and it is obtained by visualizing the true positive rate (TPR) versus the false positive rate (FPR), this last one is calculated following Equation (12):

(12) $$FPR=1-Specificity=\frac{FP}{FP+TN}$$

- Area under the curve (AUC):

Once a ROC curve is generated, the area under such curve can also be used to measure the performance of the algorithm. The AUC shows the probability that a randomly classified positive sample becomes a higher score than a randomly classified negative sample. It can be expressed as shown in Equation (13):

(13) $$AUC=P(X_{pos}>X_{neg})$$

- Matthews correlation coefficient (MCC):

Typically used to evaluate the performance of classifiers when dealing with data with an unbalanced proportion of elements in each of the labels of the target variable [46], it is obtained by following Equation (14):

(14) $$MCC=\frac{\left(TP*TN\right)-\left(FP*FN\right)}{\sqrt{\left(TP+FP\right)\left(TP+FN\right)\left(TN+FP\right)\left(TN+FN\right)}}$$

## 4. Results

As shown in Section 3.2, each eye fundus image is represented by a vector of 30 topological descriptors and, then, given to a machine learning classification algorithm to predict image quality for clinical use.

Table 1 shows the resulting topological descriptors after the extraction and vectorization of the 30 topological indicators per image, as explained in Section 3.2.

Preliminary explorations for the selection of a suitable algorithm were done on six classification algorithms (classifiers). Table 2 shows the performance metrics of the algorithms used in this phase. From these results, three algorithms were selected for the next steps in the process: SVM, MLP, and LoGit.

In the following step, the authors performed a fine-tuning of hyperparameters of the three selected algorithms. Table 3 shows the resulting performance metrics after this process. Finally, the algorithm selected for the classification task was a LoGit because of the robust performance metrics it yielded and the relatively low computational cost when compared with both SVM and MLP.

Table 4 shows the hyperparameters and values used for the tuning process for LoGit. The optimal results were obtained when tolerance = 1 × 10^−8^, C = 150,000, solver = liblinear, and maximum iterations = 10,000.

**Figure 11:**
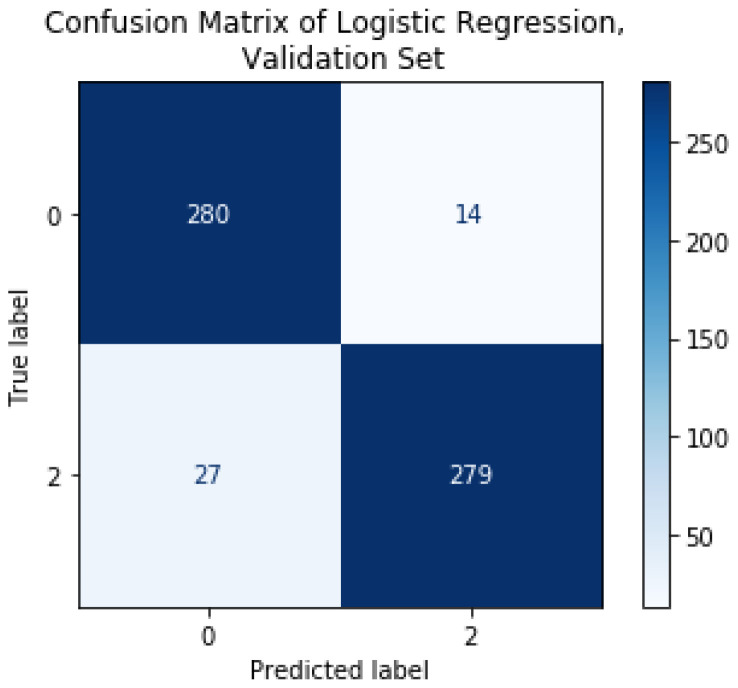
Confusion matrix of LoGit classification results on validation subset of 600 images.

**Figure 12:**
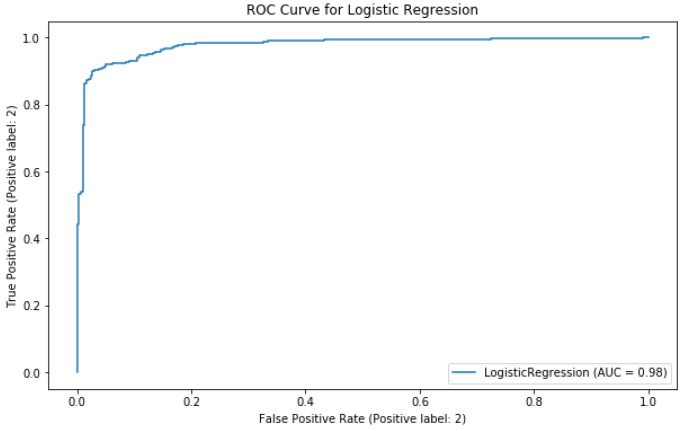
Receiver-operating characteristic curve for LoGit classification performance on validation subset of 600 images.

Once the LoGit was trained, it was tested on 600 images it had not previously classified. The results show consistency in the classification within classes as well as the global classification accuracy, as shown in Table 5. The Matthews correlation coefficient is also acceptable at 0.864, indicating consistent results in all the confusion matrix categories, as shown in Figure 11. Figure 12 shows the corresponding ROC curve.

**Figure 13:**
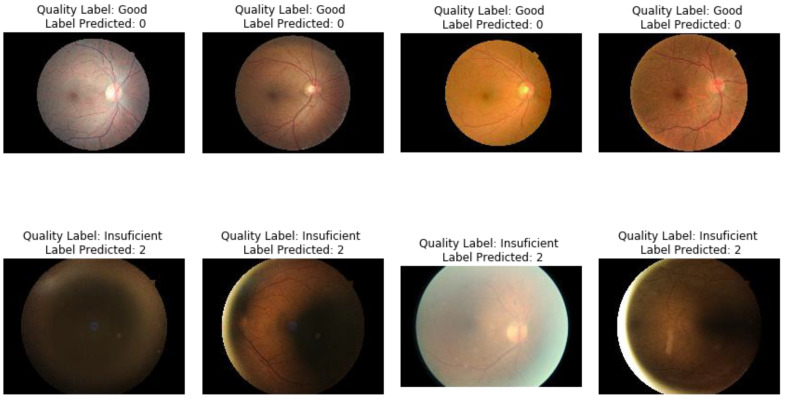
Receiver-operating characteristic curve for LoGit classification performance on validation subset of 600 images.

**Figure 14:**
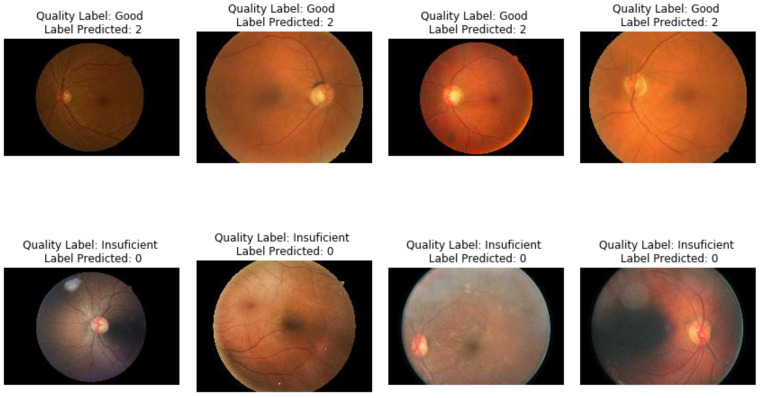
Subset of images wrongly classified by LoGit algorithm when compared to ground truth labels. For labels predicted value 0 = good, value 2 = bad.

In order to bridge the numeric results to the clinical practice in the task of image quality assessment of eye fundus, a visualization of images adequately classified when compared to ground truth labels is presented in Figure 13. Figure 14 presents examples of images where LoGit wrongly classified them according to the ground truth.

On these visualizations, it is apparent that the topological descriptors are robust to changes in color, illumination, and blurring, despite being vectors conformed by 30 elements, all derived from vectorizations of representations of persistence diagrams.

## 5. Discussion

Digital fundoscopy has become a valuable tool in the ophthalmic toolkit of contemporary clinical practice. With the advent of telemedicine and electronic medical records [47], fundoscopy has moved to the mobile arena where now it is possible to perform it via smartphones [5,48]. With these relatively new approaches reaching the clinical practice, computer-aided diagnosis systems have also been proposed to assist the clinical process in situ or remotely [49,50].

One of the first steps in the digital fundus image analysis pipeline is the quality assessment of the image obtained, as presented in Section 1.2. This preprocessing step functions as a triage station allowing us to filter out those images not meeting the minimum quality needed to continue for clinical use [16,19,21,24]. This step is also performed by the clinician, but it is trivial for humans, given how the brain processes and understands images. For machines, this step is fundamental and not trivial, since it will allow a smoother process down the pipeline of a CAD system.

As mentioned in Section 1.2, IQA techniques can be classified into three general groups, each representing the state of development in digital image analysis at the time of their publications (image characteristics, segmentation, and deep learning). To the knowledge of the authors, this is the first work using topological data analysis (TDA) to tackle the challenge of IQA in eye fundus images. The advantages of using TDA is that it represents less computational burden to the system, given that each image is first interpreted as a cubical complex and a cubical persistence calculation obtained represented by persistence diagrams, from which topological descriptors are extracted and vectorized. This allows us to run the classification task not on an image matrix but on a vector much smaller than the input data. Therefore, TDA, for the context of IQA in eye fundus images, contributes to an inherent dimensionality reduction in the data. This fact makes the method attractive, especially for contexts where limited computational power is of significance, such as when clinicians integrate mobile devices or telemedicine to their practice.

Another beneficial contribution is that the topological descriptors seem to be able to capture enough data from the image that a relatively simple and computationally not demanding algorithm suffices to render results with robust classification metrics; this has also been observed by [51]. Figure 13 shows evidence to the idea presented in the previous sentence, where the images in the examples present variability in their color, illumination, blurring, and anatomical landmarks. Still, the topological descriptors encapsulate sufficient information for the classifier to perform robustly. For the instances where the classification is not appropriate, it seems that the localization of the optic nerve, as well as a significant proportion of irregularity in color distribution in the same image, as well as blurring could explain the errors; nevertheless, more studies are needed in this direction to better understand this phenomenon.

Lastly, TDA seems to capture information on the form of topological descriptors, which allows for the classification to perform close to the ground truth labels, which in the context of images from clinical practice such as the EyePACS project, allows for the proposition of this method capturing information close to the way a clinician recollects image characteristics in order to decide if the image is of enough quality to be integrated in the clinical attention of the patient the data come from.

## 6. Conclusions

We propose a novel method for image quality assessment of eye fundus images based on the extraction of topological descriptors integrated into a machine learning classifier. The classification metrics are robust, and there is evidence that a topological approach facilitates the interpretation of the data in a similar way to how a clinician engages in these tasks during their practice. Further work is needed to investigate in more detail the clinical interpretations that might be suitable for the topological results obtained from eye fundus images. This could facilitate the use of TDA in later stages of the computer-assisted diagnosis pipeline in computational ophthalmological approaches.

## Figures and Tables

**Table 1:** List of the 30 topological indicators calculated per image.

Variables 1–6	Variables 7–12	Variables 13–18	Variables 19–24	Variables 25–30
Persistence entropy $\beta_{0}$	*2-*Wasserstein distance $\beta_{0}$	Persistence landscape $L^{2},\;k=1,\beta_{0}$	Betti curve $L^{2},\beta_{0}$	Gaussian kernel $L^{2},\sigma =1.6,\beta_{0}$
Persistence entropy $\beta_{1}$	*2-*Wasserstein distance $\beta_{1}$	Persistence landscape $L^{2},\;k=1,\beta_{1}$	Betti curve $L^{2},\beta_{1}$	Gaussian kernel $L^{2},\sigma =1.6,\beta_{1}$
Bottleneck distance $\beta_{0}$	Persistence landscape $L^{1},\;k=1,\beta_{0}$	Persistence landscape $L^{2},\;k=2,\beta_{0}$	Gaussian kernel $L^{1},\sigma =1.6,\beta_{0}$	Gaussian kernel $L^{2},\sigma =3.2,\beta_{0}$
Bottleneck distance $\beta_{1}$	Persistence landscape $L^{1},\;k=1,\beta_{1}$	Persistence landscape $2,\;k=2,\beta_{1}$	Gaussian kernel $L^{1},\sigma =1.6,\beta_{1}$	Gaussian kernel $L^{2},\sigma =3.2,\beta_{1}$
*1-*Wasserstein distance $\beta_{0}$	Persistence landscape $L^{1},\;k=2,\beta_{0}$	Betti curve $L^{1},\beta_{0}$	Gaussian kernel $L^{1},\sigma =3.2,\beta_{0}$	Number of points in diagram $\beta_{0}$
*1-*Wasserstein distance $\beta_{1}$	Persistence landscape $L^{1},\;k=2,\beta_{1}$	Betti curve $L^{1},\beta_{1}$	Gaussian kernel $L^{1},\sigma =3.2,\beta_{1}$	Number of points in diagram $\beta_{1}$

**Table 2:** Performance metrics from classification algorithms initially evaluated.

Model	AUC	CA	Precision	Recall	F1-Score
Support Vector Machine (SVM)	0.845	0.749	0.761	0.749	0.746
Decision Tree	0.870	0.894	0.895	0.894	0.894
*k-*Nearest Neighbors (*k-*NN)	0.941	0.898	0.900	0.898	0.898
Random Forest (RFC)	0.960	0.911	0.912	0.911	0.911
Logistic Regression (LoGit)	0.974	0.925	0.925	0.925	0.925
Multilayer Perceptron (MLP)	0.981	0.935	0.935	0.935	0.935

Where AUC = area under the curve and CA = classification accuracy.

**Table 3:** Performance metrics of fine-tuned classification algorithms.

Algorithm	Precision Training Set	Precision Testing Set	Recall Training Set	Recall Testing Set	F1-Score Training Set	F1-Score Testing Set
SVM	0.961	0.957	0.961	0.957	0.961	0.957
MLP	0.910	0.930	0.910	0.930	0.910	0.930
LoGit	0.989	0.987	0.989	0.987	0.989	0.987

**Table 4:** Hyperparameters values for the tuning process of LoGit.

Parameter	Value
Tolerance	{1 × 10^−4^, 1 × 10^−6^, 1 × 10^−8^}
C	{50,000, 100,000, 150,000}
Solver	{lbfgs, saga, liblinear}
Maximum iterations	{10,000, 50,000, 100,000}

**Table 5:** Classification report of LoGit with subset of not previously seen images.

Label	Precision	Recall	F1-Score	Classification Accuracy	Count
good	0.912	0.952	0.932	0.932	294
bad	0.952	0.912	0.932		306

Matthews correlation coefficient: 0.864.

## Data Availability

For this study the publicly available eyePACS dataset was used. This data can be found here: https://www.kaggle.com/c/diabetic-retinopathy-detection/data.
